# Exploratory Research on the Urine Proteome in Patients With Diabetic Peripheral Neuropathy

**DOI:** 10.1155/ije/6652078

**Published:** 2025-12-22

**Authors:** Lilong Wei, Yongwei Jiang, Haitong Wang, Jianqiang Wu, Chengwu Han, Yuliang Zhan, Yun Zhou, Yongtong Cao, Youhe Gao

**Affiliations:** ^1^ Clinical Laboratory, China-Japan Friendship Hospital, Beijing, 100029, China, zryhyy.com.cn; ^2^ Gene Engineering Drug and Biotechnology Beijing Key Laboratory, College of Life Sciences, Beijing Normal University, Beijing, 100871, China, bnu.edu.cn; ^3^ Beijing No. 159 High School, Beijing, 100035, China; ^4^ Institute of Clinical Medicine, National Infrastructure for Translational Medicine, State Key Laboratory of Complex Severe and Rare Diseases, Peking Union Medical College Hospital, Chinese Academy of Medical Sciences & Peking Union Medical College, Beijing, 100730, China, cams.ac.cn

**Keywords:** diabetes, disease biomarkers, neuropathy, peripheral nerve injury, urinary proteomics

## Abstract

Diabetic peripheral neuropathy (DPN) is a common complication of diabetes and there are currently no biomarkers for DPN in clinical practice. The objective of this study was to explore the diagnostic value of urine for diseases and discover potential markers. Urinary proteomics was used to analyze urine samples from patients with DPN and healthy individuals. Quantitative analysis of mass spectrometry data was performed using both group‐based and paired individual analysis strategies. A total of 2611 proteins were identified, of which 1759 were quantifiable, and 312 proteins showed significant differences (fold change > 2, adjusted *p* value < 0.01), with 239 decreased and 73 increased. A random grouping cross‐validation strategy was employed to evaluate the differentially expressed proteins, ensuring that the probability of random differences was less than 10%. Unsupervised clustering analysis of urinary proteomes among groups could distinguish most specimens. The most enriched GO biological processes were cytoplasmic translation, translation, exocytosis, chaperone‐mediated protein folding and immunoglobulin‐mediated immune response. The most significant KEGG signaling pathways were ribosome, coronavirus disease (COVID‐19), and metabolic pathways. Furthermore, some differentially expressed proteins were linked to the pathogenesis of diabetes, whereas others were associated with nerve damage. These molecules could serve as potential diagnostic and therapeutic biomarkers; some differential proteins have not yet been reported to be associated with DPN, providing clues for further research on their functions.

## 1. Introduction

The number of diabetes cases worldwide is rising quickly, creating a major medical challenge [1]. Diabetic peripheral neuropathy (DPN) is a common complication of diabetes. Additionally, neuropathic pain is often challenging to treat and negatively impacts their quality of life [2, 3]. Early identification and preventive measures are essential in managing DPN. Clinically, DPN is defined as the presence of symptoms and/or signs of neuropathy in individuals with prediabetes or diabetes, after ruling out other causes of peripheral neuropathy [4, 5]. Despite numerous studies on the mechanisms of DPN, the exact pathophysiology remains unclear [6]. Nerve conduction tests and electromyography have higher sensitivity than clinical examination in assessing peripheral symmetric polyneuropathy and are the minimally variable noninvasive measurement methods for neuropathy and its progression. However, nerve conduction studies are labor‐intensive and costly, making their implementation in routine clinical care impractical [7, 8]. There is still a lack of effective diagnostic markers in clinical practice.

The advancement of proteomics technology enables a thorough identification of disease markers [9, 10], with urine recognized as the most noninvasive source in clinical research [11]. An exploratory study of urinary proteome was performed in trigeminal neuralgia, which provided new directions for immune‐related treatment of the disease and potential disease markers [12]. To date, no studies have used urinary proteomics technology to investigate diseases related to DPN. This study aims to explore DPN using urinary proteomics for the first time. It seeks to assess the research potential of urinary proteomics in this context and to identify urinary markers associated with nerve injury.

## 2. Materials and Methods

### 2.1. Experimental Design and Urine Sample Collection

This study was approved by the Ethics Committee of the China‐Japan Friendship Hospital (Approval No. 2023‐KY‐126). Participants were recruited from patients and health check‐up individuals at the China‐Japan Friendship Hospital. Sample collection took place from February 2023 to June 2023. Patients with DPN were enrolled based on the clinical criteria [13]: documented history of diabetes, typical symptoms of peripheral nerve pain, test for clinical signs of DPN, such as pinprick sensation, temperature threshold sensation, vibration perception, 10°g force test, and exclusion of pain attributable to other causes. Patients with tumor diseases, kidney diseases, or other neurological conditions were excluded. Urine proteomics technology was used to quantitatively analyze urine proteomes from both healthy individuals and those with DPN. The study included urine samples from 11 healthy individuals (7 males, 4 females; age range 32–71, mean 50.7, standard deviation 10.8) and 9 patients with DPN (6 males, 3 females; age range 41–74, mean 55.8, standard deviation 11.7). All samples were collected after clinical testing and were stored at −80°C after collection.

### 2.2. Handling of Urine Samples

Urine proteins were precipitated using ethanol [14], and then protein from each sample was digested with the filter‐aided sample preparation (FASP) method [15], specifically as follows: the collected urine samples were centrifuged at 12, 000 × g for 30 min at 4°C, and the supernatant was transferred to a 50 mL centrifugal tube. Dithiothreitol solution (DTT, Sigma) was then added to a final concentration of 20 mM, and the mixture was shaken and incubated in a water bath at 37°C for 1 h before being cooled to room temperature. Iodoacetamide (IAA, Sigma) was added to a final concentration of 50 mM, and the mixture was shaken and reacted in the dark at room temperature for 40 min. Six times the volume of precooled absolute ethanol was added, and the mixture was homogeneously mixed and precipitated at −20°C for 24 h. On the second day, the mixture was centrifuged at 4°C, 12, 000 × g for 30 min, and the supernatant was discarded. The protein precipitate was resuspended in lysis buffer (containing 8 mol/L urea, 2 mol/L thiourea, 25 mmol/L dithiothreitol, and 50 mmol/L Tris). After centrifugation at 12, 000 × g for 30 min at 4°C, the supernatant was placed in a new EP tube. The protein concentration was measured by the Bradford method. Urine protein digestion: 100 μg urine protein sample was added to the filter membrane of 10 kDa ultrafiltration tube (Pall, Port Washington, NY, USA) and placed in an EP tube, and 25 mmol/L NH_4_HCO_3_ solution was added to make the total volume 200 μL. Then, the membrane washing operation was carried out: ① 200 μL UA solution (8 mol/L urea, 0.1 mol/L Tris‐HCl, pH 8.5) was added and centrifuged and washed twice at 14,000×g 5 min 18°C; ② loading: the sample was added and centrifuged at 14,000×g 40 min 18°C; ③ 200 μL UA solution was added and centrifuged at 14, 000 × g for 40 min at 18°C, repeated 2 times; ④ 25 mmol/L NH_4_HCO_3_ solution was added and centrifuged at 14, 000 × g 40 min 18°C for 3–4 times; ⑤ trypsin (Trypsin Gold, Promega, Fitchburg, WI, USA) was added at a ratio of 1:50 for digestion, and the water bath was kept at 37°C for 12–16 h. The next day, the peptide segment was collected by centrifugation at 13, 000 × g 30 min 4°C and passed through the HLB column (Waters, Milford, MA, USA) for desalting. The eluent was vacuum dried at 4°C for approximately 1.5 h. The peptide segments were then collected and stored at −80°C.

### 2.3. LC‐MS/MS Tandem Mass Spectrometry Analysis

The digested samples were dissolved in 0.1% formic acid and quantified using the BCA kit. Equivalent peptide amount was loaded. The peptide concentration was diluted to 0.5 μg/μL. Four μL of each sample was taken to prepare the mixed polypeptide sample, and the separation was performed using a high pH reversed phase peptide separation kit (Thermo Fisher Scientific) according to the instructions. Ten fractions were collected by centrifugation, and after drying using a vacuum dryer, they were resuspended in 0.1% formic acid. The iRT reagent (Biognosys, Switzerland) was added at a sample: iRT volume ratio of 10:1 to calibrate the retention time of the extracted peptide peaks. For analysis, 1 μg of peptides from each sample was taken, and mass spectrometry analysis and data acquisition were performed using an EASY‐nLC 1200 chromatography system (Thermo Fisher Scientific, USA) and an Orbitrap Fusion Lumos Tribrid mass spectrometer (Thermo Fisher Scientific, USA). In order to generate the spectral library, the separated 10 fractions were analyzed by mass spectrometry in data dependent acquisition (DDA) mode. The mass spectrometry data were collected in high sensitivity mode. A complete mass spectrometry scan was obtained in the range of 350–1500 m/z with a resolution setting of 60,000. Individual samples were analyzed using the data independent acquisition (DIA) mode. DIA acquisition was performed using a DIA method with 36 windows. After every 8 samples, a single DIA analysis of pooled peptides was performed as quality control [16].

### 2.4. Database Search and Label‐Free DIA Quantification

The raw data (RAW files) collected from the liquid chromatography‐mass spectrometry were imported into Proteome Discoverer (version 2.1, Thermo Scientific), using the SwissProt database (taxonomy: *Homo*; containing 20,346 sequences). The data were matched, and the iRT sequences were added to the database. The search results were then imported into Spectronaut Pulsar (Biognosys AG, Switzerland) for processing and analysis. The abundance of peptides was calculated by summing the peak areas of the respective fragment ions in MS2. The protein intensity was calculated by summing the peptide abundances to determine protein abundance.

### 2.5. Data Analysis

Each sample was performed in triplicate, and all three values were used for statistical analysis. The identified proteins were compared, and differentially expressed proteins were screened. The loose screening criteria for differentially expressed proteins were fold change (FC) ≥ 1.5 or ≤ 0.67 and adjusted *p* value < .05 by two‐tailed unpaired *t*‐test analysis. The strict screening criteria for differentially expressed proteins were FC ≥ 2 or ≤ 0.5 and adjusted *p* value < 0.01 by two‐tailed unpaired *t*‐test analysis. To further evaluate the reliability of differentially expressed proteins, the probability of random generation of differentially expressed proteins was calculated by random grouping using the average value of each protein. All samples were randomly grouped to identify differentially expressed proteins, and then the ratios of the differential proteins generated under different random methods to that in the experiment were calculated to evaluate the reliability of the results. Functional enrichment analysis of the screened differentially expressed proteins was performed using the Wukong platform (https://www.omicsolution.org/wkomic/main/), Uniprot website (https://www.uniprot.org/), and DAVID database (https://david.ncifcrf.gov/). The reported literature was retrieved from the PubMed database (https://pubmed.ncbi.nlm.nih.gov) for functional analysis of differentially expressed proteins.

## 3. Results

### 3.1. Comparative Analysis of Urinary Proteomics Differential Proteins

LC‐MS/MS proteomic analysis was conducted on urine samples collected from 11 healthy individuals (N) and 9 patients with DPN. There were no differences in age and gender between the two groups of participants (details in the supporting information (available here)). A total of 2611 proteins were identified with at least one specific peptide and protein‐level false discovery rate (FDR) less than 1%. Following data processing, 1759 proteins were available for quantitative analysis. Correlation analysis was performed between patients with DPN and healthy control samples (Figure 1). PCA analysis showed that the quality control samples were evenly distributed at the center of the results. OPLS‐DA analysis effectively distinguished between DPN and healthy controls. Unsupervised clustering analysis of the identified urinary proteins from DPN and healthy controls showed a certain degree of aggregation for both groups, while some patients were found to be close to healthy controls. The volcano plot from the quantitative analysis showed many significantly different proteins between the two groups.

Figure 1Visualization analysis of identified proteins. (a) Principal component analysis (PCA) of QC, DPN, and N; (b) orthogonal signal correction‐based partial least squares discriminant analysis (OPLS‐DA); (c) unsupervised clustering analysis of the urine proteome of diabetic peripheral lesion patients and healthy controls; and (d) volcano plot display.

**Figure:**
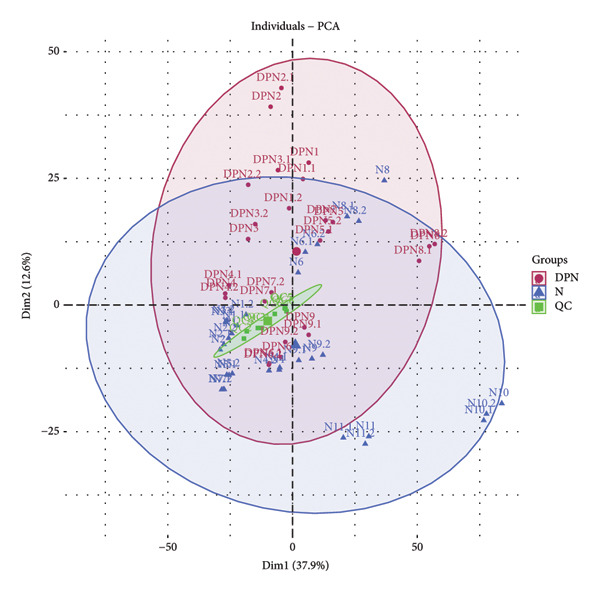
(a)

**Figure:**
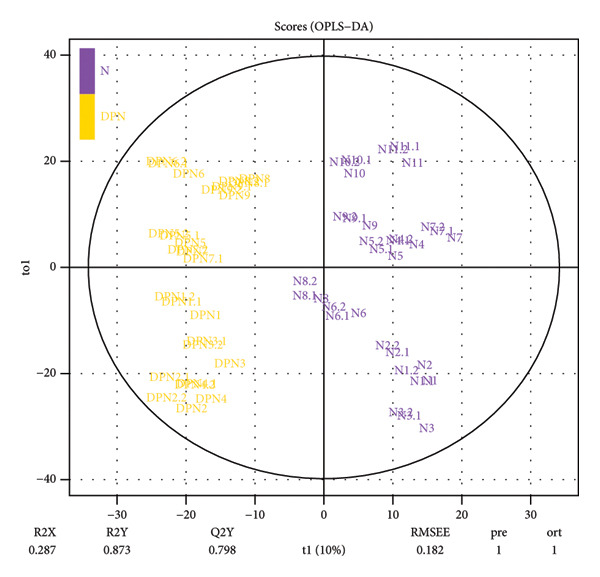
(b)

**Figure:**
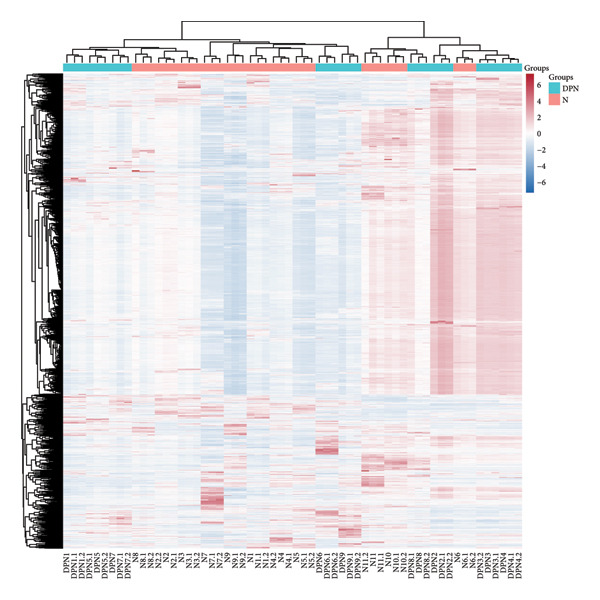
(c)

**Figure:**
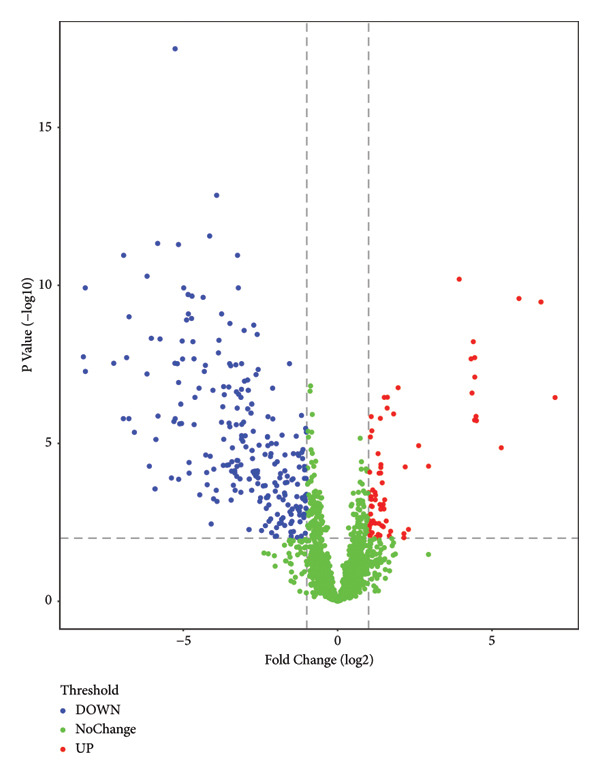
(d)

### 3.2. Differential Protein Analysis

The differences between two experimental specimens were compared, with screening conducted under both relaxed and strict conditions. The number of differential proteins identified between different groups is shown in Table 1. To further evaluate the reliability of the differential proteins, a random grouping method was used to calculate the likelihood of the differential proteins being randomly generated. Specifically, a total protein identification was conducted by randomly selecting 11 healthy samples and 9 samples from patients with DPN (FC ≥ 1.5 or ≤ 0.67, *p* < 0.05), resulting in an average of 68.3 differential proteins, indicating that at least 74.8% of the differential proteins were not randomly generated; under stricter conditions (FC ≥ 2 or ≤ 0.5, *p* < 0.01), random grouping validation yielded an average of 7.35 differential proteins, indicating that at least 90.7% of the differential proteins were not randomly generated (Table 1).

**Table 1:** Differential protein credibility in random grouping cross‐validation analysis.

Group	Differential protein screening criteria	Differential protein quantity	Randomly generated number of differential proteins	Differential protein credibility
DPN vs N	FC ≥ 1.5 or ≤ 0.67; *p* < 0.05	271	68.3	0.748
	FC ≥ 2 or ≤ 0.5; *p* < 0.01	79	7.35	0.907

*Note:* N, healthy individuals.

Abbreviations: DPN, diabetic peripheral neuropathy; FC, fold change.

### 3.3. Functional Analysis

The differential proteins generated under strict selection criteria have a reliability of over 90% in random group experimental validation. To enhance the reliability of the data, the significantly different proteins were analyzed using adjusted *p* value, and the functional analysis of the differential proteins (fold change > 2, adjusted *p* value < 0.01) was conducted. GO biological processes and KEGG signaling pathway analysis have been performed online using the David database; a total of 19 biological processes (*p* < 0.01) and 10 signaling pathways (*p* < 0.01) were enriched in the differential proteins between the two groups, as shown in Table 2.

**Table 2:** Differential protein GO functional analysis and KEGG analysis.

Category	Term	Count	%	*p* value	Fold enrichment
GO	Cytoplasmic translation	16	5.03	0.000	11.41
	Translation	22	6.92	0.000	6.36
	Exocytosis	11	3.46	0.000	5.82
	Chaperone‐mediated protein folding	7	2.20	0.000	10.43
	Immunoglobulin‐mediated immune response	9	2.83	0.000	5.57
	Protein folding	11	3.46	0.000	4.10
	Cell–cell adhesion	11	3.46	0.001	3.68
	SNARE complex disassembly	3	0.94	0.001	49.19
	Protein transport	18	5.66	0.002	2.38
	Vesicle‐mediated transport	12	3.77	0.002	3.04
	Intracellular protein transport	12	3.77	0.004	2.83
	Protein stabilization	11	3.46	0.005	2.93
	Osteoblast differentiation	8	2.52	0.005	3.86
	Chaperone‐mediated protein folding independent of cofactor	3	0.94	0.006	24.59
	Regulation of exocytosis	4	1.26	0.007	10.09
	Positive regulation of telomere maintenance via telomerase	4	1.26	0.007	10.09
	Positive regulation of telomerase RNA localization to Cajal body	3	0.94	0.008	21.86
	Epidermis development	6	1.89	0.008	4.86
	Positive regulation of protein localization to Cajal body	3	0.94	0.010	19.67
	Cytoplasmic translation	16	5.03	0.000	11.41

KEGG_PATHWAY	Ribosome	17	5.35	0.000	4.66
	Coronavirus disease (COVID‐19)	19	5.97	0.000	3.76
	Metabolic pathways	57	17.92	0.000	1.71
	Proteasome	7	2.20	0.000	7.17
	Biosynthesis of nucleotide sugars	6	1.89	0.001	7.65
	Parkinson’s disease	15	4.72	0.002	2.61
	Necroptosis	11	3.46	0.002	3.26
	Carbon metabolism	9	2.83	0.003	3.66
	Spinocerebellar ataxia	10	3.14	0.003	3.27
	Biosynthesis of amino acids	7	2.20	0.005	4.40

*Note:* N, healthy individuals.

Abbreviations: DPN, diabetic peripheral neuropathy; GO, gene ontology; KEGG, Kyoto Encyclopedia of Genes and Genomes.

### 3.4. Individualized Analysis of Urinary Proteomics

In order to better assess the disease status of each patient with DPN and achieve precise diagnosis and treatment, we conducted a one‐to‐many comparative analysis between individual patient samples and 11 samples from a healthy group. The different proteins screened under relaxed conditions and their expression trends are shown in Table 3. There was a significant number of different proteins between DPN and healthy controls, which is notably higher than the number of different proteins found in group protein analysis. The individualized analysis shows considerable differences between different patients and healthy controls.

**Table 3:** Individualized comparison of urinary proteomics (FC ≥ 1.5 or ≤ 0.67, adjusted *p* value < 0.05).

Group	Trends in differential protein changes	Sample number/number of significantly different proteins								
		1	2	3	4	5	6	7	8	9
DPN vs N	Total	770	883	871	1066	678	816	713	664	652
	↓	402	424	403	449	389	397	323	406	355
	↑	368	459	468	617	289	419	390	258	297

*Note:* N, healthy individuals.

Abbreviations: DPN, diabetic peripheral neuropathy; FC, fold change.

To clarify whether there were common differential proteins among patients with the same group of diseases, we analyzed the differential proteins screened under relaxed conditions from DPN specimens and healthy controls. In the DPN group, 10 differential proteins were consistently identified in all 9 specimens, and 16 proteins were commonly identified in 8 specimens, showing a relatively consistent trend in expression changes (Table 4).

**Table 4:** High‐confidence differential protein screening between individual specimens and healthy controls (FC ≥ 1.5 or ≤ 0.67, adjusted *p* value < 0.05).

UniProt entry	Protein name	Trend of change	Proportion	Protein annotation link
Q3LXA3	Triokinase/FMN cyclase	↓	9/9	https://www.uniprot.org/uniprotkb/Q3LXA3/entry#function
Q13057	Bifunctional coenzyme A synthase	↓	9/9	https://www.uniprot.org/uniprotkb/Q13057/entry
Q00796	Sorbitol dehydrogenase (SDH)	↓	8/9	https://www.uniprot.org/uniprotkb/Q00796/entry
P13473	Lysosome‐associated membrane glycoprotein 2 (LAMP‐2)	↓	8/9	https://www.uniprot.org/uniprotkb/P13473/entry
O95954	Formimidoyltransferase cyclodeaminase	↓	8/9	https://www.uniprot.org/uniprotkb/O95954/entry
P56385	ATP synthase subunit e, mitochondrial	↓	8/9	https://www.uniprot.org/uniprotkb/P56385/entry
P61254	Large ribosomal subunit protein uL24	↓	8/9	https://www.uniprot.org/uniprotkb/P61254/entry
Q16819	Meprin A subunit alpha	↑	9/9	https://www.uniprot.org/uniprotkb/Q16819/entry
O60240	Perilipin‐1	↑	9/9	https://www.uniprot.org/uniprotkb/O60240/entry
P20142	Gastricsin	↑	9/9	https://www.uniprot.org/uniprotkb/P20142/entry
A0A0A0MT36	Immunoglobulin kappa variable 6D‐21	↑	9/9	https://www.uniprot.org/uniprotkb/A0A0A0MT36/entry
A0A075B6R9	Probable nonfunctional immunoglobulin kappa variable	↑	8/9	https://www.uniprot.org/uniprotkb/A0A075B6R9/entry
P01743	Immunoglobulin heavy variable 1–46	↑	8/9	https://www.uniprot.org/uniprotkb/P01743/entry
Q96P63	Serpin B12	↑	8/9	https://www.uniprot.org/uniprotkb/Q96P63/entry
P12110	Collagen alpha‐2 (VI) chain	↑	8/9	https://www.uniprot.org/uniprotkb/P12110/entry
Q9UHG0	Doublecortin domain‐containing protein 2	↑	8/9	https://www.uniprot.org/uniprotkb/Q9UHG0/entry
Q16661	Guanylate cyclase activator 2B	↑	8/9	https://www.uniprot.org/uniprotkb/Q16661/entry
O00526	Uroplakin‐2 (UP2)	↑	8/9	https://www.uniprot.org/uniprotkb/O00526/entry
P20231	Tryptase beta‐2 (Tryptase‐2)	↑	8/9	https://www.uniprot.org/uniprotkb/P20231/entry
P35556	Fibrillin‐2	↑	8/9	https://www.uniprot.org/uniprotkb/P35556/entry
P0DMQ5	Putative transmembrane protein INAFM2	↑	8/9	https://www.uniprot.org/uniprotkb/P0DMQ5/entry
P05164	Myeloperoxidase (MPO)	↑	8/9	https://www.uniprot.org/uniprotkb/P05164/entry

Abbreviation: FC, fold change.

## 4. Discussion

### 4.1. Urinary Proteomics Can Effectively Display the Characteristics of Different Patients With DPN

This study was the first to use urinary proteomics to investigate patients with DPN. By visually presenting the urinary proteomics of DPN patients and healthy controls, urinary proteomics could effectively distinguish between different populations (Figure 1). Unsupervised clustering analysis of the urinary proteome from both groups shows that most specimens from the same group cluster together, while some individuals exhibit similarities with specimens from other groups. This is likely due to individual differences.

### 4.2. Functional Analysis of Differential Proteins Between Groups

The selection of differential proteins between different groups was conducted under both relaxed and strict conditions. To evaluate the accuracy of the selection results, a random grouping method was used to calculate the likelihood of differential proteins being randomly generated, thereby assessing the probability that the proteins were not randomly generated but resulted from true differences in the samples. Data in Table 1 indicate selecting strict conditions for screening results in an overall reliability of over 90% for the differential proteins. The random grouping method for calculating differential proteins fully considered the differences between individual samples, further assessing the reliability of the data.

In group analysis, adjusted *p* values were used to analyze significantly different proteins. Compared to healthy controls, DPN enriched a total of 19 biological processes and 10 signaling pathways (Table 2). Two biological processes were related to Cajal bodies; Cajal bodies are nuclear organelles that contain factors necessary for splicing, ribosome biogenesis, and transcription, participating in various key biological processes, including the biogenesis of snRNPs and snoRNPs, mRNA decay, and gene silencing [17]. Research has shown that telomerase RNA can bind to signals directing its localization to Cajal bodies and exert its effects [18, 19]. At the same time, animal experiments have found that sensory neurodegeneration in diabetes involves changes in nuclear structure and function, especially unique alterations in the spliceosome, with an increased number of Cajal bodies [20]. In our study, the biological process of positive regulation of telomerase RNA localization to the Cajal body was significantly enriched in patients with DPN. Whether a telomerase–Cajal body regulatory mechanism contributes to disease onset requires further validation.

Seven biological processes were related to protein translation, folding, stability, transport, and degradation. Immunoglobulin‐mediated immune responses were significantly enriched, indicating that immune responses are involved in the disease’s occurrence, consistent with other studies [21–23]. In the KEGG signaling pathway analysis, metabolic pathways were significantly enriched in DPN patients; fructose and mannose metabolisms are associated with the onset of type 2 diabetes, which is consistent with the patients being diabetic [24]. However, whether these changes are involved in the occurrence of DPN needs further verification.

### 4.3. High‐Confidence Differential Proteins in Individual Samples

In the experiment, analysis was conducted using individual samples compared to a healthy control group. Under relaxed screening conditions, the identified differential proteins accounted for one‐third or more of the total quantified proteins and were nearly twice as many as those found in group analysis, indicating that individual differences were common and have a significant impact in the experiment. Therefore, identifying protein molecules with common characteristics through individualized analysis is more credible. In this experiment, a total of 22 high‐confidence proteins were identified, of which appeared in at least 8 of the 9 patients.

Among the significantly different proteins, some were found to be involved in metabolic changes related to the onset of diabetes, while others were associated with nerve damage. Additionally, some proteins were found that have not been reported to be related to relevant diseases, providing clues for studying molecular functions.

In the DPN group, triokinase/FMN cyclase is involved in fructose metabolism, responsible for phosphorylating glyceraldehyde to glyceraldehyde‐3‐phosphate. Fructose plays an important role in metabolic diseases [25]; triokinase/FMN cyclase may be related to the onset of diabetes. Formimidoyltransferase cyclodeaminase has been shown to be associated with diabetes [26]. The significantly different protein molecules mentioned above suggest a possible association with the disease group related to diabetes patients.

ATP synthase subunit e, mitochondrial, is a multifunctional enzyme complex involved in ATP generation, and literature reports that mutations in this enzyme can lead to peripheral neuropathy [27]. Sorbitol dehydrogenase (SDH) is a key enzyme in the polyol pathway, which interconverts glucose and fructose through sorbitol, an important alternative pathway for glucose metabolism. The polyol pathway is believed to be related to the etiology of hyperglycemia‐induced diabetic complications, such as diabetic neuropathy and retinopathy [28]. Doublecortin domain‐containing protein 2 is widely distributed, highly expressed in the brain, inhibits the classical Wnt signaling pathway [29], and is related to reading ability and the regulation of neurodevelopment [30]. Fibrillin‐2 is essential for the development of peripheral nerves, myelination, and regeneration [31]. The aforementioned protein molecules are elevated in the disease group, which is presumed to be related to neuropathy.

Bifunctional coenzyme A synthase, a bifunctional enzyme, catalyzes the fourth and fifth consecutive steps of the coenzyme A biosynthesis pathway and may play a regulatory role in coenzyme A biosynthesis. Its abnormalities can lead to various pathological changes, such as diabetes and neurodegeneration [32]. Lysosome‐associated membrane glycoprotein 2 (LAMP‐2) plays an important role in lysosome biosynthesis, lysosomal pH regulation, and autophagy. LAMP‐2–dependent fusion and degradation processes of autophagosomes are involved in the pathogenesis of obesity‐related diabetes [33]. Probable nonfunctional immunoglobulin kappa variable, immunoglobulin heavy variable 1–46, and immunoglobulin kappa variable 6D‐21 are involved in antigen recognition during immune responses [34], confirming the presence of inflammatory responses in DPN [6]. Myeloperoxidase (MPO) plays an important role in neuro‐related inflammation [35, 36]. Changes in related molecules may be involved in the pathogenesis of complex diseases or reveal the mechanisms of disease onset.

Meprin A subunit alpha, a membrane‐bound oligomeric metalloproteinase, has been reported to be associated with kidney damage; this study focuses on diabetic patients, which may be related to diabetic nephropathy and requires further validation. Large ribosomal subunit protein uL24 is a large ribonucleoprotein complex responsible for intracellular protein synthesis; perilipin‐1 is involved in metabolic activities including severe familial partial lipodystrophy and early‐onset acute coronary syndrome; serpin B12 inhibits trypsin and plasmin but does not inhibit thrombin, coagulation factor Xa, or urokinase‐type plasminogen activator; additionally, collagen alpha‐2 (VI) chain, gastricsin, guanylate cyclase activator 2B, uroplakin‐2, tryptase beta‐2 (tryptase‐2), and putative transmembrane protein INAFM2 also showed significant changes. These molecules were found for the first time to have significant differences in the urine of patients with DPN compared to the normal population, providing clues for studying their related functions.

At the same time, we also noted significant differences in the discoidin, CUB, and LCCL domain‐containing protein 2 (DCBLD2) in group analysis. DCBLD2 is a neurofibrillary protein‐like transmembrane scaffold receptor that has known and expected roles in vascular remodeling and neuronal localization, and it is also upregulated in certain tumors [37], which may make it a potential disease‐related biomarker.

Diabetes has many complications that often coexist, with diabetic nephropathy being one of the most common. Several differential proteins identified in our study were related to diabetic nephropathy, such as meprin A, which was significantly increased compared to the control group in all the DPN. However, there was no difference in the comparison between blood creatinine and glomerular filtration rate. This highlights the significant role of urine proteomics in studying complex diseases, and it is likely to have more clinical significance in the early stages of the disease. Improving the specificity of urine markers for different complications requires further research. In the future, refined disease screening or combining urine proteomics with tissue proteomics association studies may yield better results.

### 4.4. Diagnostic Advantages of Urine Proteomics in DPN

Urine is the final metabolic product of blood that is filtered through the glomerulus and then reabsorbed, excreted, and secreted in the renal tubules and collecting ducts. Changes in the composition, quantity, and characteristics of urine provide information about the occurrence, development, and prognosis of urinary system diseases. They also reflect the overall metabolic status of the body [38, 39]. Most of the disease‐related differential proteins identified in this experiment were derived via the tissue‐blood‐urine pathway. DPN is a common complication of diabetes. Early diagnosis is beneficial for effective disease management. Neural conduction testing is currently the most commonly used method for detecting nerve damage. However, this approach is invasive and inefficient, making it unsuitable for disease screening and monitoring. One of the biggest advantages of urine is its noninvasiveness, and there are no restrictions on urine sample collection, making it easy to monitor diseases, which is incomparable to nerve conduction testing. The results of this experiment demonstrate the diagnostic potential of urine proteomics for DPN. The identified nerve damage‐related molecules were used as potential markers for screening and monitoring.

### 4.5. Limitations of the Experiment

This experiment is the first attempt to use urinary proteomics technology to identify differences between patients with DPN and healthy individuals. Due to limitations in conditions, the sample size of the experiment is relatively small. Nevertheless, we attempted to evaluate the experimental results using a one‐to‐many strategy while applying stricter selection criteria to identify differential proteins, ensuring the reliability of the results. DPN is a complex disease that encompasses not only the pathophysiological processes of diabetes onset but also the related pathophysiological processes of neuropathy, which increases the complexity of the experiment. The identified differential proteins reflected differences arising from both diabetes and neuropathy. Due to limitations in conditions, the subgroups of diabetes without complications, diabetes with nephropathy, and diabetes with retinopathy were not compared. In the future, increasing the sample size and further refining the grouping of the disease may yield more comprehensive results. As this is an exploratory study, key differential proteins have not yet been independently validated, such as enzyme‐linked immunosorbent assay (ELISA) and Western blot. Large‐scale sample size or clinical cohort studies are needed in the future to clarify the application value of these key markers.

## 5. Conclusion

This study is the first to comprehensively present DPN using urinary proteomics, providing an in‐depth description of the urinary proteomic characteristics in patients with DPN and revealing potential pathogenic mechanisms that may exist in this patient population. Some significantly different protein molecules have been reported for the first time in DPN, offering broader clues for studying molecular functions. Additionally, high‐confidence proteins related to the disease were identified in the experiment, which could serve as potential disease markers for diagnosis and treatment.

## Ethics Statement

The study was approved by the Ethics Committee of China‐Japan Friendship Hospital (approval number: 2023‐KY‐126) and implemented in strict accordance with the relevant ethics standards and the Declaration of Helsinki.

## Disclosure

All authors have checked and approved the final version of the manuscript.

## Conflicts of Interest

The authors declare no conflicts of interest.

## Author Contributions

Lilong Wei: project administration and writing–original draft preparation. Yongwei Jiang: methodology; Haitong Wang: investigation and formal analysis. Jianqiang Wu: mass spectrometry analysis. Chengwu Han: specimen collection: Yuliang Zhan: formal analysis. Yun Zhou: funding acquisition. Yongtong Cao: methodology and project administration. Youhe Gao: conceptualization, funding acquisition and writing–review and editing. Lilong Wei, Yongwei Jiang, and Haitong Wang contributed equally to this study and share the first authorship. Youhe Gao, Yuliang Zhan, and Yongtong Cao are corresponding authors.

## Funding

This study received support from the National Key R&D Program of China (2023YFA1801901), Beijing Natural Science Foundation (L246002), and National High‐Level Hospital Clinical Research Funding (2023‐NHLHCRF‐YYPPLC‐TJ‐27).

## Supporting Information

The experiment’s relevant data can be found in the Supporting Information. It includes five main worksheets, which are detailed as follows:

Worksheet 1: protein data, which primarily contains intensity values from mass spectrometry for each sample.

Worksheet 2: *p* < 0.01; significant differential proteins with FC > 2.

Worksheet 3: data analysis table, which includes the intensity values of three replicated mass spectrometry identifications for each specimen.

Worksheet 4: details of GO analysis data.

Worksheet 5: details of KEGG signaling pathway data.

## Supporting information


**Supporting Information** Additional supporting information can be found online in the Supporting Information section.

## Data Availability

The data that support the findings of this study are available in the Supporting Information of this article.
